# Supplementary material for: Facile synthesis of Fe-doped ZIF-8 and its adsorption of phosphate from water: Performance and mechanism
Source: PLoS One. 2024 Oct 11;19(10):e0311239. doi: 10.1371/journal.pone.0311239 (PMC12140080; doi:10.1371/journal.pone.0311239)
Supplement: S1 File — (DOCX) [file pone.0311239.s001.docx]

**Facile synthesis of Fe-doped ZIF-8 and its adsorption of phosphate from water: Performance and mechanism**

*Zhijia Miao^1,2,3,4^, Xueqiang Song^1,2^, Xiaolei Wang^3^, Hao Wang^4^, Shuoyang Li^2,3^, Zhen Jiao^1,2,3,4*^*

*1 Hebei Center for Ecological and Environmental Geology Research, Hebei GEO University, Shijiazhuang 050031, China*

*2 School of Water Resources and Environment, Hebei GEO University, Shijiazhuang 050031, China*

*3 Wastewater Treatment and Resource Reusing Technology Innovation Center of Hebei Province, Hebei Yuehai Water Group Co., Ltd., Shijiazhuang 050031, China.*

*4 Norendar International Co., Ltd., Shijiazhuang 050031, China.*

* Corresponding author: Zhen Jiao

E-mail: Jiaozhen2012@126.com

**Adsorption equations:**

The adsorption amount (*q*_t_, mg/g) and equilibrium adsorption amount (*q*_e_, mg/g) were calculated from Eqs. (1) and (2):

$q_{t}=\frac{(C_{0}-C_{t})\times V}{m}$ (1)

$q_{e}=\frac{(C_{0}-C_{e})\times V}{m}$ (2)

where *C*_0_, *C*_t_, and *C*_e_ (mg/L) are the initial concentration, the concentration at time *t*, and the equilibrium concentration of phosphate ions in the solution, respectively. *V* (mL) represents the volume of the cesium solution, and *m* (g) is the mass of the adsorbent.

To explore the mechanism of mass transfer, two typical kinetic models, i.e., the pseudo-first-order model (PFO) and pseudo-second-order model (PSO), were used to simulate experimental data using the following equations:

Pseudo-first-order model

$\ln\left( q_{e}-q_{t} \right)=lnq_{e}-k_{1}t$ (3)

Pseudo-second-order model

$\frac{t}{q_{t}}=\frac{1}{k_{2}q_{e}^{2}}+\frac{1}{q_{e}}t$ (4)

In Eqs. (3) and (4), *q*_t_ and *q*_e_ (mg/g) represent the adsorption amounts of phosphate at time *t* and the equilibrium adsorption amount of phosphate after reaching equilibrium time. *k*_1_ (h^-1^), and *k*_2_ (g/(mg h)) are the rate constants in the pseudo-first-order and pseudo-second-order equations (Eqs. (3) and (4)), respectively.

Two known isothermal models were adopted to fit the experimental data, i.e., the Langmuir model and the Freundlich model (Eqs. (5) and (6)), with the following linear equations:

Langmuir model：

$\frac{C_{e}}{q_{e}}=\frac{C_{e}}{q_{m}}+\frac{1}{K_{L}q_{m}}$ (5)

Freundlich model：

$logq_{e}=logK_{F}+\frac{1}{n}logC_{e}$ (6)

where *C*_e_ (mg/L) and *q*_e_ (mg/g) are the equilibrium concentration of phosphate and its corresponding equilibrium adsorption capacity, respectively. *K*_L_ (L/mg) and *K*_F_ (mg/g) (L/mg)^1/n^ represent the constants of the Langmuir model and the Freundlich model, respectively.
