# Supplementary material for: Facile synthesis of Fe-doped ZIF-8 and its adsorption of phosphate from water: Performance and mechanism
Source: PLoS One. 2024 Oct 11;19(10):e0311239. doi: 10.1371/journal.pone.0311239 (PMC12140080; doi:10.1371/journal.pone.0311239)
Supplement: Attachment — Submitted filename: comments.docx [file pone.0311239.s003.docx]

I think the manuscripts well. But, my questions is for FTIR spectra? Why only for 1400 cm-1, usually until 4000 cm (wavenumbers). Please revise the FTIR spectra. I think O-H group also influence for adsorption process.
